# Comments from the departing Editor

**DOI:** 10.1098/rstb.2022.0471

**Published:** 2023-01-30

**Authors:** John Pickett

My term as an Editor comes to an end with the last issue of 2022. I was fortunate to inherit a highly successful journal programme from my predecessor, Dame Linda Partridge FRS, and I am delighted to be able to pass the Editorship into the very capable hands of Prof. Richard Dixon FRS. I am sure that he will find working for the journal as exciting and rewarding as I have.

*Philosophical Transactions of the Royal Society B* is a unique journal, and it has been an interesting experience understanding and developing the role that this journal represents for the academic community, as well as the challenges that a journal of this nature faces in today's publishing environment. Great satisfaction as Editor derives from the prestige with which the journal continues to be regarded across the scientific and wider academic communities. Furthermore, I believe that there is clear evidence of the high regard of currently practising life scientists for the journal from the great commitment that we see from our Guest Editors, authors and reviewers. Publishing 26 theme issues a year on schedule could not be achieved without the journal's Guest Editors, authors and reviewers, and I would like to use this opportunity to thank everyone who has contributed during the last 7 years, and particularly since 2020 during the potentially very diverting viral pandemic.

One of the most enjoyable aspects of being the Editor of a journal with such a broad scope has been in seeing the great range of topics that are submitted as ideas for theme issues. The Editor also plays a crucial role in determining the programme for the flagship scientific meetings that the Royal Society holds every year (as these are automatically published in the journal), which provides an insight into emerging research areas. I personally find that the issues which are published in association with these discussion meetings are often pioneering in expressing new ideas emerging during the discussion among experts at all stages of scientific development and particularly from those in the early stages of their careers.

During the last 7 years, we have published an extremely broad range of topics across all areas of biology; from molecular structure to climate change and neuroscience to disease modelling. Some of our most-cited articles during this time have come from issues relating the environment. ‘The interaction of fire and mankind’ (https://royalsocietypublishing.org/toc/rstb/2016/371/1696) was a theme issue that arose from a Royal Society Discussion Meeting. More recently ‘Climate change and ecosystems: threats, opportunities and solutions’ (https://royalsocietypublishing.org/doi/10.1098/rstb.2019.0104) came from a joint National Academy–Royal Society meeting. Both of these issues have proved highly influential and have been discussed widely. Articles from the area of neuroscience have also been widely cited, including articles on ‘interoception’ [1], empathy across species [2] and sex differences in sleep [3]. I found a theme issue on ‘The political brain’ (https://royalsocietypublishing.org/toc/rstb/2021/376/1822), published in 2021, particularly fascinating. However, with a note of regret, the success of the climate change-related theme issue reminded me that a proposal went to the Hooke committee in June 2017 for a Discussion Meeting on ‘The Nitrogen Challenge’ which would have drawn together experts from a broad spectrum of both the physical and life sciences. Both the Editor of *Philosophical Transactions of the Royal Society A* and I attended a planning meeting with the organizers and expressed great enthusiasm at the prospect of publishing a joint, or two companion, themed issue(s) focused on this subject now regarded as a major challenge to mitigating emissions of ‘greenhouse’ gases. Discussions were at the time initiated with the journals' Commissioning Editors to explore ways in which the papers could appear in a single issue of *Philosophical Transactions*. However, although by a democratic process without question, the Hooke committee decided against supporting the Discussion Meeting considered to be essential for the joint *A* and *B* theme issue and the project was abandoned. I am nonetheless greatly encouraged by the success of the climate change-related theme issue mentioned above and that *Philosophical Transactions B* now publishes on average three ‘climate change’ issues every year with more planned for the future.

It has been a great pleasure to watch the journal go from strength to strength and retain its high status in the publishing landscape. Article downloads have increased from under 7 million in 2016 to 11 million per year in 2021 (this may be partly owing to a change in website host and reporting practices, but we know that there has been significant growth in that time). Our readers come from all around the world. This growth in readership probably corresponds, at least in part, to the growth in Open Access during the last few years; in 2022 over 50% of articles are being published under an Open Access (OA) model, making them immediately free to read. The introduction of the Read & Publish library subscription model has played a large part in driving this; authors at institutions with one of these deals can publish OA at no cost to themselves as OA fees are covered by a deal with the institution.

Other changes during my time as Editor have related to the growing and, in my view, highly commendable ‘open science’ movement, such as the establishment of open data policies and the growth in use of preprint servers. It is been a fascinating time to be Editor of a prominent journal and to navigate the changing landscape. The Royal Society journal team often leads the way in adopting new practices and policies, whilst supporting our authors. For example, all of the Royal Society journals welcome the use of preprints and cover costs of data deposition in Dryad or Figshare. Partnerships with FundRef, ORCID, CRediT (Contributor Roles Taxonomy) and Web of Science Reviewer Recognition all help authors and reviewers to gain credit for their contributions.

During the last few years, we have been working hard to increase Guest Editor, author and reviewer diversity on the journal. In 2021, almost 25% of our authors were from outside Western Europe and North America, compared to just 11% in 2015. Overall, 65 countries were represented in 2021 (44 in 2015). We actively encourage contributions from early career scientists (ECRs); and it has been great to work with several young Guest Editors recently. It can be a fantastic way for ECRs to grow their networks and gain editorial experience, with the support that the journal Editorial Office provides during the process. Our Editorial Board is tactically selected to represent a broad range of countries, subject areas and career stages. I am very pleased that over 50% of our Board in 2022 are women.

Although I have come to an end of my time on *Philosophical Transactions B*, I will be continuing to follow its activities and I look forward to seeing what interesting subjects are covered in the future. Also, I hope to contribute directly myself as I have done during my Editorship and since 1985. I would strongly encourage those with a good idea for a topic to submit a proposal; it can be both a very effective vehicle for promoting an emerging field of research, but also an extremely rewarding and, I believe, enjoyable experience for the Guest Editors.

Finally, I would like to thank both the Editorial Board and the Royal Society's Publishing staff for their efficiency, professionalism and creativity, which has made my job both productive and extremely enjoyable. My thanks in particular go to Helen Eaton who has been the Commissioning Editor during my term. It has been a great pleasure to work with her and to experience the innovation by which she has continually ensured my attention during periods of high demand on my time from other commitments. Helen has been key to the growing success of *Philosophical Transactions B* in recent years.
